# Ultrastructural Characteristics of the Mature Spermatozoon of *Artyfechinostomum malayanum* (Digenea: Echinostomatidae), an Intestinal Parasite of *Rattus norvegicus* (Rodentia: Muridae) in Vietnam

**DOI:** 10.3390/ani14192813

**Published:** 2024-09-29

**Authors:** Abdoulaye Jacque Sacodou Bakhoum, Adji Mama Marigo, Srisupaph Poonlaphdecha, Alexis Ribas, Serge Morand, Jordi Miquel

**Affiliations:** 1 Laboratoire de Biologie Évolutive, Écologie et Gestion des Écosystèmes, Faculté des Sciences et Techniques, Université Cheikh Anta Diop de Dakar, Dakar-Fann BP 5005, Senegal; abjabak@gmail.com (A.J.S.B.); maricoadji@yahoo.fr (A.M.M.); 2 Faculté des Sciences et Technologies de l’Éducation et de la Formation, Université Cheikh Anta Diop de Dakar, Boulevard Habib Bourguiba, Dakar-Fann BP 5005, Senegal; 3 Secció de Parasitologia, Departament de Biologia, Sanitat i Medi Ambient, Facultat de Farmàcia i Ciències de l’Alimentació, Universitat de Barcelona, Av. Joan XXIII, sn, 08028 Barcelona, Spain; spoonlaphdecha@ub.edu (S.P.); aribas@ub.edu (A.R.); 4 Institut de Recerca de la Biodiversitat (IRBio), Universitat de Barcelona, Av. Diagonal, 645, 08028 Barcelona, Spain; 5 IRL HealthDEEP, CNRS—Kasetsart University—Mahidol University, Bangkok 10900, Thailand; serge.morand@umontpellier.fr; 6 Faculty of Veterinary Technology, Kasetsart University, Bangkok 10900, Thailand

**Keywords:** *Artyfechinostomum malayanum*, Digenea, Echinostomatidae, sperm characteristics, ultrastructure

## Abstract

**Simple Summary:**

The ultrastructural study of spermatozoa in parasites such as the Digenea provides data that, like classical morphological features, biological data, and genetic characterization, can help achieve a better understanding of phylogenetic relationships at different taxonomic levels. In this context, the present study provides the first ultrastructural data of spermatozoa in a digenean species of the genus *Artyfechinostomum*. These results increase the database of ultrastructural spermatological characteristics within the group of Echinostomatoidea. The comparison of these features with the existing data allows us to establish a coherent model of the sperm cell for species belonging to this superfamily.

**Abstract:**

The study of sperm characteristics has proven useful for elucidating interrelationships in several groups of Platyhelminthes, such as digeneans. Thus, in the present work, the ultrastructural organization of the mature spermatozoon of the digenean *Artyfechinostomum malayanum* (Echinostomatidae), a parasite of *Rattus norvegicus* (Rodentia: Muridae) from Dong Thap Province, Vietnam, was investigated for the first time using transmission electron microscopy. The male gamete of *A. malayanum* exhibits two axonemes of different lengths, showing the 9 + ‘1’ pattern of the Trepaxonemata, a nucleus, two mitochondria, two lateral expansions, two bundles of parallel cortical microtubules, external ornamentation, spine-like bodies, and granules of glycogen. Thus, the mature spermatozoon follows a Type V sperm model proposed for digeneans. We also highlight some noteworthy characteristics in Echinostomatidae with possible phylogenetic implications, such as two lateral expansions in the anterior region of the spermatozoon and two mitochondria.

## 1. Introduction

The family Echinostomatidae, first described by Looss in 1899, is notable for its distinct circumoral headcollar, which features one or two crowns of large spines interrupted ventrally [1]. Some species within this family cause diseases in humans, domestic animals, and wildlife, while others serve as important model organisms in research [2,3]. The life cycle of echinostomatids involves three hosts: one definitive host and two intermediate hosts. Aquatic snails act as the first intermediate hosts, while various species of snails, clams, frogs, and fish serve as the second intermediate hosts, carrying the encysted metacercaria. Humans and other definitive hosts typically become infected by consuming these second intermediate hosts raw or undercooked [4]. Within the Echinostomatidae, several genera including *Acanthoparyphium*, *Artyfechinostomum*, *Echinochasmus*, *Echinoparyphium*, *Echinostoma*, *Himasthla*, *Hypoderaeum*, and *Isthmiophora* pose significant epidemiological risks due to their potential to infect humans [4,5,6]. *Artyfechinostomum malayanum* has been reported in humans in Eastern Asia (China, India, Indonesia, Lao PDR, Malaysia, Philippines, Singapore, and Thailand). Snails of the genera *Gyraulus* and *Indoplanorbis* serve as the first intermediate hosts and *Gyraulus*, *Indoplanorbis*, and *Pila* species as the second intermediate hosts of this echinostomatid. Moreover, diverse animals such as dogs, cats, rats, pigs, mice, hamsters, and house shrews act as reservoirs [7].

The evolutionary relationships and classification of the family Echinostomatoidae are intricate, as it seems to be a polyphyletic taxon divided into three major, well-supported clades [3].

To elucidate the phylogeny and classification of the subclass Digenea, researchers have investigated spermatological characteristics to uncover features that could be valuable for interpreting the complex relationships within this taxonomic group [8,9,10,11,12].

In the superfamily Echinostomatoidea, which comprises 17 families and 105 genera [13], studies of sperm ultrastructure remain limited [14]. Detailed analyses are available for only four species from two families: the fasciolids *Fasciola hepatica* and *Fasciola gigantica* [15,16], and the echinostomatids *Echinostoma caproni* [17] and *Hypoderaeum conoideum* [14]. Additionally, only one micrograph in the literature depicts the sperm cells of another echinostomatid, *Echinostoma togoensis* [18].

In this study, we aim to provide the first detailed data on the spermatozoon of *Artyfechinostomum malayanum* (Leiper, 1911) (Echinostomatidae) using transmission electron microscopy. Moreover, we perform a comparative analysis of the ultrastructural characteristics of the previously unexplored genus *Artyfechinostomum* with the available results, which is crucial for expanding the existing database and improving our understanding of relationships within the superfamily Echinostomatoidea.

## 2. Materials and Methods

### 2.1. Specimens

Adult specimens of *A. malayanum* were freshly collected from the intestine of a brown rat, *Rattus norvegicus* (Berkenhout, 1769) (Rodentia: Muridae), captured in April 2013 in Dong Thap Province, Vietnam (10°26′45.96″ N, 105°41′9.6″ E). Detailed methods for sample collection are described elsewhere [19]. Rodents were investigated for several zoonotic pathogens within the Wellcome Trust-funded project Vietnam Initiative for Zoonotic Infections (VIZIONS) (WT/093724). The rodents’ guts were collected for further investigations, including of parasitic helminths. Rat trapping was carried out with the agreement of the sub-Department of Animal Health in Dong Thap Province. The rodents were humanely culled with an overdose of an inhalant anesthetic (isoflurane) following American Veterinary Medical Association (AVMA) guidelines [20].

### 2.2. Transmission Electron Microscopy

Specimens were fixed in cold (4 °C) 2.5% glutaraldehyde in a 0.1 M sodium cacodylate buffer (pH 7.4) for at least 2 h and then rinsed in the same buffer. Then, they were postfixed in cold (4 °C) 1% osmium tetroxide in the buffer for 1 h, rinsed in Milli-Q water (Millipore Gradient A10, Millipore Co., Merck KGaA, Darmstadt, Germany), and dehydrated through an ethanol series and propylene oxide. They were embedded in Spurr resin and polymerized at 60 °C for 72 h. Ultrathin sections were cut with a Reichert-Jung Ultracut-E ultramicrotome (Leica Microsystems, Wetzlar, Germany), placed on copper grids, and double-stained with uranyl acetate and lead citrate using Reynolds’ method [21].

Gold grids were prepared to detect the presence of glycogen using Thiéry’s technique [22]. The procedure involved treating the grids with periodic acid, thiocarbohydrazide, and silver proteinate (PA-TCH-SP) as follows: 30 min in 10% PA, rinsing with Milli-Q water, 24 h in TCH, further rinsing in acetic solutions and Milli-Q water, 30 min in 1% SP in the dark, and final rinsing in Milli-Q water. All ultrathin sections were then examined with a JEOL 1010 transmission electron microscope (JEOL Ltd., Tokyo, Japan) operated at 80 kV at the “Centres Científics i Tecnològics” of the University of Barcelona (CCiTUB).

## 3. Results

**Figure 1:**
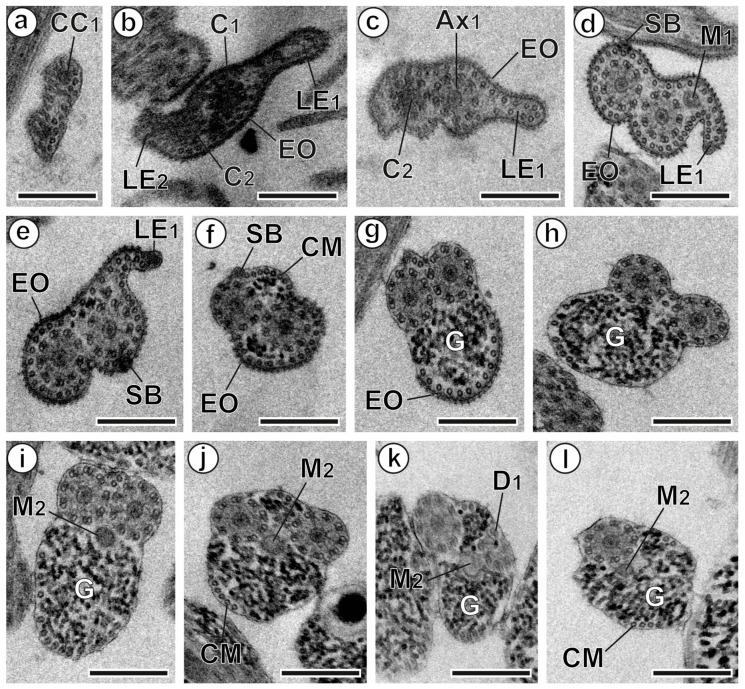
Mature spermatozoon of *Artyfechinostomum malayanum* (Regions I and II). (**a**–**c**) Consecutive cross-sections of the anterior extremity of the spermatozoon, highlighting the presence of lateral expansions. (**d**,**e**) Cross-sections of Region I, showing both axonemes fully formed, external ornamentation of the plasma membrane, spine-like bodies, and cortical microtubules. Note the position of the first mitochondrion adjacent to the first lateral expansion. (**f**,**g**) Cross-sections of the posterior area of Region I, displaying external ornamentation, spine-like bodies, and cortical microtubules. (**h**) Cross-section of the anterior part of Region II. (**i**,**j**) Cross-sections showing the appearance of the second mitochondrion. (**k**,**l**) Correlative cross-sections demonstrating the progressive disappearance of the first axoneme. (Ax_1_) first axoneme; (C_1_ and C_2_) centrioles of the first and second axonemes; (CC_1_) central core of the first centriole; (D_1_) doublets of the first axoneme; (EO) external ornamentation of the plasma membrane; (G) granules of glycogen; (LE_1_ and LE_2_) first and second lateral expansions; (M_1_ and M_2_) first and second mitochondrion; (SB) spine-like bodies. Scale bars = 300 nm.

**Figure 2:**
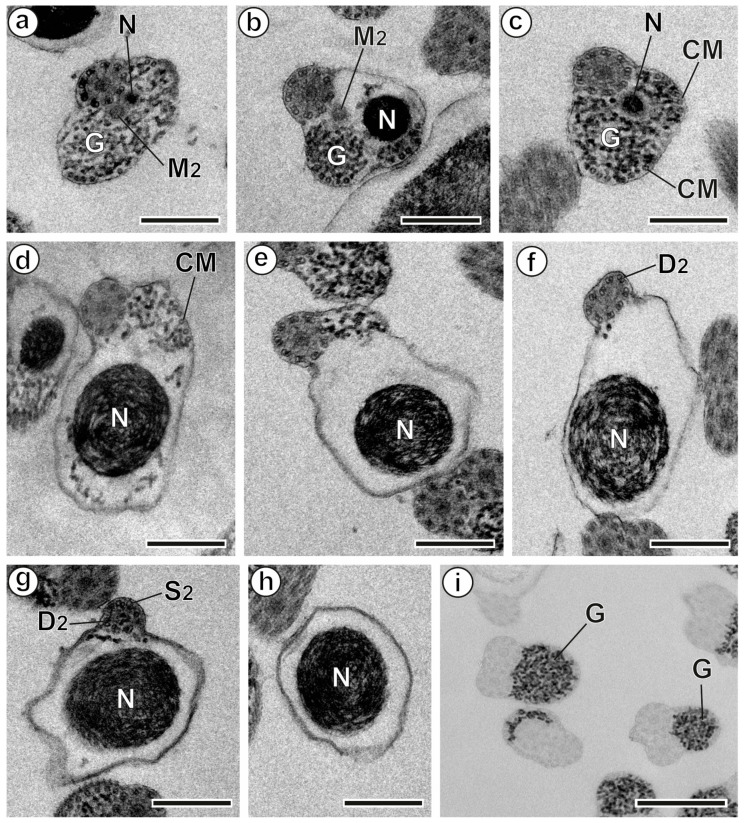
Mature spermatozoon of *Artyfechinostomum malayanum* (Region III) and Thiéry’s test. (**a**,**b**) Cross-sections in the anterior part of Region III, showing the simultaneous presence of the second mitochondrion and the nucleus. (**c**–**h**) Correlative cross-sections of Region III, illustrating the sequence of changes towards the posterior extremity of the spermatozoon. Note the progressive reduction in the number of cortical microtubules, the disorganization of the second axoneme, and the presence of only the nucleus at the posterior tip. (**i**) Thiéry’s cytochemical test revealing glycogen granules. (CM) cortical microtubules; (D_2_) doublets of the second axoneme; (G) granules of glycogen; (M_2_) second mitochondrion; (N) nucleus; (S_2_) singlets of the second axoneme. Scale bars (**a**–**h**) = 300 nm; (**i**) = 500 nm.

**Figure 3:**
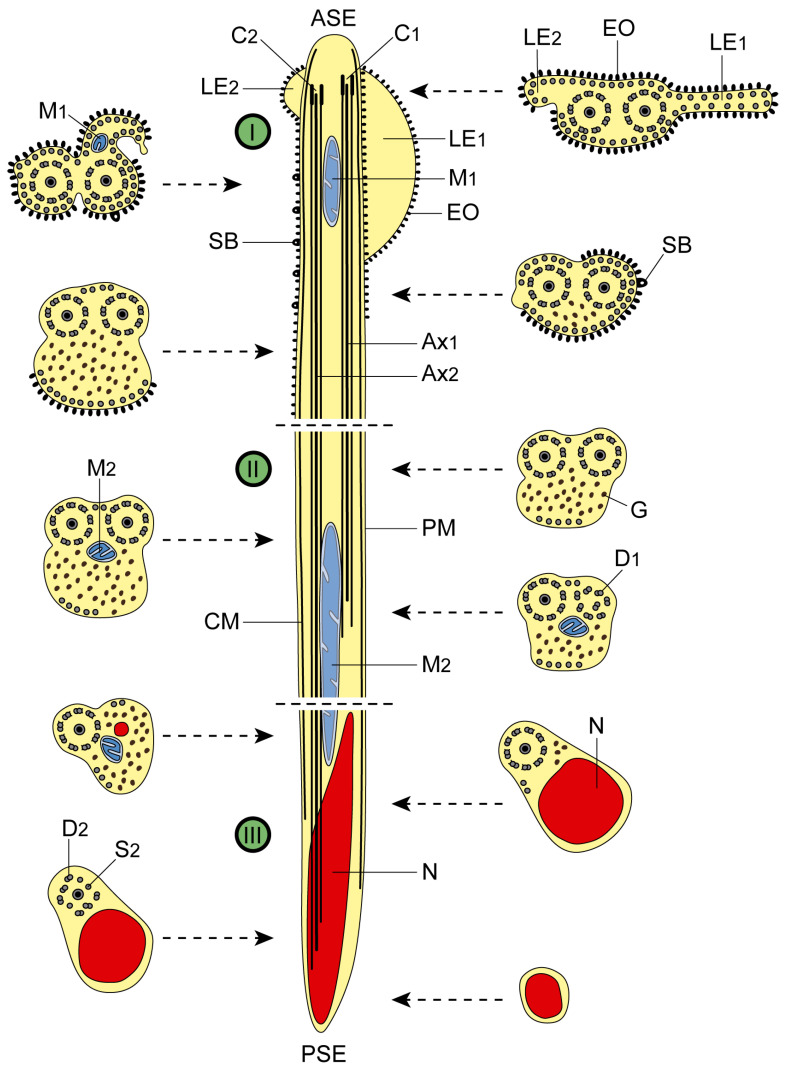
Schematic drawing of the spermatozoon of *Artyfechinostomum malayanum*. To enhance clarity, glycogen granules are omitted from the longitudinal sections. (**I**) anterior region; (**II**) middle region; (**III**) posterior region. (ASE) anterior spermatozoon extremity; (Ax_1_ and Ax_2_) first and second axoneme; (C_1_ and C_2_) centrioles of the first and second axoneme; (CM) cortical microtubules; (D_1_ and D_2_) doublets of the first and second axoneme; (EO) external ornamentation of the plasma membrane; (G) granules of glycogen; (LE_1_ and LE_2_) first and second lateral expansion; (M_1_ and M_2_) first and second mitochondrion; (N) nucleus; (PM) plasma membrane; (PSE) posterior spermatozoon extremity; (S_2_) singlets of the second axoneme; (SB) spine-like bodies.

The mature *A. malayanum* spermatozoon is a filiform cell with tapered ends. It exhibits several ultrastructural features common to digeneans, particularly within the superfamily Echinostomoidea. These include two axonemes of varying lengths with the 9 + ‘1’ trepaxonematan pattern, parallel cortical microtubules, an externally ornamented plasma membrane, spine-like bodies, two lateral expansions, a nucleus, two mitochondria, and glycogen granules. Analysis of multiple longitudinal and cross-sections reveals three distinct regions (I–III) (Figure 1, Figure 2 and Figure 3).

### 3.1. Anterior Region or Region I

The anterior end of the spermatozoon contains the central core of the first axoneme giving rise to the first centriole (Figure 1a). As the second centriole develops, cross-sections reveal two lateral expansions and an external ornamentation of the plasma membrane, accompanied by a continuous layer of cortical microtubules (Figure 1b). Moving posteriorly through Region I, when both axonemes are fully formed, subsequent cross-sections display the first mitochondrion adjacent to the second lateral expansion. This area also shows the external ornamentation of the plasma membrane, cortical microtubules, and spine-like bodies (Figure 1c–e and Figure 3I). The peak number of cortical microtubules (approximately 45) is observed here (Figure 1d). Further posterior in Region I, the lateral expansions and the first mitochondrion are no longer present. Cross-sections in this area now only reveal the two axonemes, two bundles of cortical microtubules, spine-like bodies, and an external ornamentation of the plasma membrane on one side, along with some glycogen granules (Figure 1f,g and Figure 3I).

### 3.2. Middle Region or Region II

In the anterior section of this region, both axonemes are present, along with two bundles of cortical microtubules and glycogen granules (Figure 1h and Figure 3II). Moving to the middle section, the second mitochondrion appears, while the first axoneme starts to disappear and doublets become disorganized (Figure 1i–k and Figure 3II). Notably, the maximum number of microtubules decreases compared to Region I, with about 14 observed in the region. Finally, the cross-section of the posterior part of this region reveals the second mitochondrion, one axoneme, cortical microtubules, and glycogen granules (Figure 1l and Figure 3II).

### 3.3. Posterior Region or Region III

The nucleus emerges in the posterior end of the spermatozoon. In the anterior part of Region III, the second mitochondrion is still visible in cross-sections (Figure 2a,b and Figure 3III). As the second mitochondrion disappears, the nucleus enlarges, and the number of cortical microtubules decreases until they are no longer detectable (Figure 2c–e and Figure 3III). Progressing towards the posterior part of Region III, cross-sections reveal the following sequence: the cortical microtubules and the second axoneme vanish, while the nucleus extends to the posterior tip of the mature spermatozoon (Figure 2e–h and Figure 3III).

Additionally, the granular material identified in the mature spermatozoon of *A. malayanum* was confirmed to be glycogen using Thiéry’s cytochemical test (Figure 3I).

## 4. Discussion

The spermatozoon of *A. malayanum* exhibits a general morphology typical of most digeneans, with exceptions including schistosomes and some didymozoids [23,24,25]. This mature spermatozoon features two axonemes with the 9 + ‘1’ pattern characteristic of Trepaxonemata [26], alongside two bundles of parallel cortical microtubules, lateral expansions, external ornamentation of the plasma membrane, spine-like bodies, mitochondria, a nucleus, and glycogen granules. Notably, the distinct features observed between the anterior and posterior extremities of the mature spermatozoa are particularly valuable for systematic and phylogenetic studies.

### 4.1. Anterior Region

#### 4.1.1. Anterior Spermatozoon Characters

The mature *A. malayanum* spermatozoon has two centrioles in its anterior tip, corresponding to both axonemes, as well as external ornamentation and lateral expansions (see Table 1). A similar morphology has been reported in *E. caproni* and *H. conoideum* [14,17]. However, in the latter, external ornamentation and lateral expansion appear when both axonemes are completely formed. In *Echinostoma togoensis*, the only available micrograph of the mature spermatozoon shows axonemes and two bundles of cortical microtubules [18]. Thus, no data are available on the rest of the mature spermatozoon characteristics. In other echinostomatoidean species such as the fasciolids *F. hepatica* and *F. gigantica*, a single axoneme is present in the anterior extremity of the male gamete [15,16]. Although further studies on the morphology of the anterior spermatozoon characters are needed in Echinostomatoidea, it could be an interesting feature for phylogenetic studies.

#### 4.1.2. Lateral Expansion

Lateral expansions are a notable feature observed in the anterior region of spermatozoa in various digenean species [11]. This characteristic is commonly linked with cortical microtubules, external plasma membrane ornamentation, and spine-like bodies, as seen in the sperm cells of *A. malayanum* and other echinostomatoidean species (Table 1). In this study, we identified two lateral expansions in the mature spermatozoon of *A. malayanum*. This observation aligns with findings in *H. conoideum* [14] and several other Mesometridae family members, such as *Mesometra brachycoelia* and *Wardula capitellata* [27,28]. In contrast, the fasciolids *F. hepatica* and *F. gigantica* and the echinostomatid *E. caproni* typically exhibit only one lateral expansion in their spermatozoa [15,16,17]. Additionally, a single lateral expansion has been documented in various digenean spermatozoa with different morphologies (see Bakhoum et al. [11] for a review). According to these authors, the presence of a lateral expansion is a key feature distinguishing Type V mature spermatozoon across the superfamilies Echinostomatoidea, Microscaphidioidea, Paramphistomatoidea, and Prononocephaloidea.

#### 4.1.3. Association of ‘External Ornamentation–Cortical Microtubules’ and Its Location

In male *A. malayanum* gametes, external ornamentation of the plasma membrane is found in the anterior region and is closely associated with cortical microtubules. This pattern is consistent across all echinostomatoidean species studied to date (see Table 1). This external ornamentation is specifically present in regions where the sperm exhibits lateral expansion, where the maximum number of cortical microtubules (approximately 45) can be observed. Several authors have extensively discussed the significance of this association for phylogenetic analysis. Quilichini et al. [10] classified spermatozoa into three types based on the location of external ornamentation: the first type has ornamentation in the anterior region; the second type has ornamentation situated more posteriorly; and the third type lacks ornamentation altogether. Additionally, Bakhoum et al. [11] highlighted that the presence or absence of external ornamentation in conjunction with cortical microtubules could be crucial for elucidating relationships within Digenea. Interestingly, some digenean species do not show this association. For instance, external ornamentation does not appear to be linked with cortical microtubules in faustulids [29], hemiurids [30], lecithasterids [31], sclerodistomids [32,33], and sclerodistomoids [34].

#### 4.1.4. Spine-like Bodies

Since their initial description [35], spine-like bodies have been commonly observed in mature digenean spermatozoa [11]. However, these structures may have been misinterpreted as fixation artifacts or overlooked in earlier studies (e.g., Figures 6 and 7 for *Paragonimus ohirai* in Orido [36] and Figures 10 and 11 for *Haematoloechus* in Justine and Mattei [37]).

In the superfamily Echinostomatoidea, spine-like bodies have been documented in the two fasciolid species studied so far (*F. gigantica* and *F. hepatica* [15,16]) and in the echinostomatids *H. conoideum* [14] and *A. malayanum* (Table 1). Conversely, *E. caproni* lacks these structures [17]. Additionally, spine-like bodies have been absent in all hemiuroidean species examined to date [30].

The presence or absence of spine-like bodies in digenean spermatozoa, including their occurrence within the same family and their periodicity, could serve as significant ultrastructural criteria for phylogenetic analysis [38,39,40].

#### 4.1.5. Maximum Number of Cortical Microtubules and Its Location

The classification of digenean spermatozoa based on the location of the maximum number of cortical microtubules was initially proposed by Quilichini et al. [29] and has gained broad acceptance. According to several authors [11,41], the positioning of these microtubules can vary among digenean species, making it a potentially valuable ultrastructural criterion, especially considering their role in sperm mobility. In *A. malayanum*, the maximum number of cortical microtubules (approximately 45) is situated in the anterior region of the spermatozoon. This pattern is consistent across all echinostomatoidean species described to date (see Table 1).

### 4.2. Middle Region

#### Number of Mitochondria

In male *A. malayanum* gametes, two mitochondria were observed: one in the ornamented zone and the other in the transitional area just before the nuclear region, extending into the anterior part of the nuclear area. A similar arrangement of two mitochondria has been reported in other Echinostomatoidea species within the family Echinostomatidae, except for *E. caproni* [17], which has been noted to have only one mitochondrion, as seen in fasciolid species [15,16] (Table 1).

Traditionally, digenean spermatozoa were described as containing a single mitochondrion [42]. However, recent evidence strongly supports the presence of multiple mitochondria in the mature spermatozoa of Digenea [43].

Additionally, mitochondrial morphology varies significantly among digenean species. For example, some species exhibit a moniliform mitochondrion, such as *Aphallus tubarium*, *Holorchis micracanthum*, *Macvicaria obovata*, *Opechona bacillaris*, and *Stephanostomoides tenuis* [40,44,45,46,47]. Recently, Kacem et al. [48] described a unique U-shaped mitochondrion in the male gametes of *Allopodocotyle tunisiensis*.

### 4.3. Posterior Region

#### Posterior Spermatozoon Extremity

The posterior extremity of the *A. malayanum* spermatozoon consists solely of the nucleus, representing a terminal characteristic of this species. This morphological feature is also observed in the echinostomatid *H. conoideum* and the fasciolids *F. hepatica* and *F. gigantica* [14,15,16] (Table 1). There is currently no information on the terminal characteristics of the mature *E. caproni* spermatozoon [17].

Quilichini et al. [9] categorized three types of posterior spermatozoon extremities in Digenea based on the arrangement of terminal structures: (i) Type 1 (Opecoelidean Type) characterized by the sequence ‘axoneme, nucleus, and cortical microtubules’; (ii) Type 2 (Fasciolidean Type) defined by the sequence ‘cortical microtubules, axoneme, and nucleus’; and (iii) Type 3 (Cryptogonimidean Type) marked by the sequence ‘cortical microtubules, nucleus, and axoneme’. Additionally, a distinct posterior spermatozoon morphology featuring ‘axonemes and mitochondrion’ has been reported in *Aponurus laguncula* [31].

The morphology of the posterior extremity of spermatozoa is crucial in elucidating the phylogenetic relationships among digeneans across various taxonomic levels.

### 4.4. Sperm Models in the Echinostomatoidea

The spermatozoa of *A. malayanum* and all other echinostomatoidean species examined to date conform to the Type V sperm model defined by Bakhoum et al. [11]. This model is characterized by two axonemes with the 9 + ‘1’ pattern of Trepaxonemata; the presence of lateral expansion; association of ‘external ornamentation–cortical microtubules’; external ornamentation located in the anterior part of the spermatozoon; two bundles of parallel cortical microtubules; maximum number of cortical microtubules situated in the anterior region; and generally one mitochondrion. Notably, within the Echinostomatidae family, there are distinctive features such as two lateral expansions in the anterior region and two mitochondria. Additionally, structures like spine-like bodies warrant further investigation to enhance our understanding of their role and variation.

## 5. Conclusions

The spermatological analysis of *A. malayanum* supports the general morphology typical of the Echinostomatoidea. This species adheres to the Type V sperm pattern outlined for digeneans. Notably, it exhibits significant phylogenetic features, such as two lateral expansions in the anterior region of the spermatozoon and two mitochondria. These findings highlight the importance of expanding spermatological studies within Digenea to better incorporate ultrastructural details into their phylogenetic framework.

## Figures and Tables

**Table 1:** Main spermatological characteristics of the superfamily Echinostomatoidea.

Families Species [Reference]	Anterior Region							Middle Region		Posterior Region	
	ASC	LE	LMCM	MCM	EO + CM	LEO	SB	BCM	M	PSC	TS
**Echinostomatidae**											
*A. malayanum* [present study]	2Ax–CM–EO–LE	+	AntS	45	+	AntA	+	2	2	N	V
*E. caproni* [17]	2Ax–CM	+	AntS	45	+	AntA	–	2	1	?	V
*E. togoensis* [18]	MD	MD	MD	MD	MD	MD	MD	2	MD	MD	?
*H. conoideum* [14]	2Ax–CM	+	AntS	45	+	AntA	+	2	2	N	V
**Fasciolidae**											
*F. gigantica* [16]	1Ax	+	AntS	44	+	AntA	+	2	1	N	V
*F. hepatica* [15]	1Ax	+	AntS	37?	+	AntA	+	2	1	N	V

(AntA) anterior part of the anterior region; (AntS) anterior part of the spermatozoon; (ASC) anterior spermatozoon characteristic; (Ax) axoneme; (BCM) number of bundles of cortical microtubules; (CM) cortical microtubules; (EO) external ornamentation of the plasma membrane; (EO + CM) association of external ornamentation–cortical microtubules; (LE) lateral expansion; (LEO) location of external ornamentation; (LMCM) location of the maximum number of cortical microtubules; (M) number of mitochondria; (MCM) maximum number of cortical microtubules; (MD) missing data; (N) nucleus; (PSC) posterior spermatozoon characteristic; (SB) spine-like bodies; (TS) type of spermatozoon [11]; (+) presence; (–) absence; (?) doubtful data.

## Data Availability

The data presented in this study are available upon request from the corresponding author. The data are not publicly available due to internal laboratory policy.
